# Estimation of the Basic Reproduction Numbers of the Subtypes H5N1, H5N8, and H5N6 During the Highly Pathogenic Avian Influenza Epidemic Spread Between Farms

**DOI:** 10.3389/fvets.2021.597630

**Published:** 2021-06-24

**Authors:** Woo-Hyun Kim, Seongbeom Cho

**Affiliations:** College of Veterinary Medicine and Research Institute for Veterinary Science, Seoul National University, Seoul, South Korea

**Keywords:** avian influenza, basic reproduction number, Korea, H5N1, H5N6, H5N8

## Abstract

It is important to understand pathogen transmissibility in a population to establish an effective disease prevention policy. The basic reproduction number (*R*_0_) is an epidemiologic parameter for understanding the characterization of disease and its dynamics in a population. We aimed to estimate the *R*_0_ of the highly pathogenic avian influenza (HPAI) subtypes H5N1, H5N8, and H5N6, which were associated with nine outbreaks in Korea between 2003 and 2018, to understand the epidemic transmission of each subtype. According to HPAI outbreak reports of the Animal and Plant Quarantine Agency, we estimated the generation time by calculating the time of infection between confirmed HPAI-positive farms. We constructed exponential growth and maximum likelihood (ML) models to estimate the basic reproduction number, which assumes the number of secondary cases infected by the index case. The Kruskal-Wallis test was used to analyze the epidemic statistics between subtypes. The estimated generation time of H5N1, H5N8, and H5N6 were 4.80 days [95% confidence interval (CI) 4.23–5.38] days, 7.58 (95% CI 6.63–8.46), and 5.09 days (95% CI 4.44–5.74), respectively. A pairwise comparison showed that the generation time of H5N8 was significantly longer than that of the subtype H5N1 (*P* = 0.04). Based on the ML model, *R*_0_ was estimated as 1.69 (95% CI 1.48–2.39) for subtype H5N1, 1.60 (95%CI 0.97–2.23) for subtype H5N8, and 1.49 (95%CI 0.94–2.04) for subtype H5N6. We concluded that *R*_0_ estimates may be associated with the poultry product system, climate, species specificity based on the HPAI virus subtype, and prevention policy. This study provides an insight on the transmission and dynamics patterns of various subtypes of HPAI occurring worldwide. Furthermore, the results are useful as scientific evidence for establishing a disease control policy.

## Introduction

Highly pathogenic avian influenza (HPAI) is a highly contagious viral disease that infects domestic poultry and wild birds (1). The HPAI virus can cause an epidemic that may spread rapidly, has a high mortality rate among domestic birds, and devastates the poultry industry (2). Outbreaks of distinct subtypes of HPAI, including H5N1, H5N8, and H5N6, are continually reported worldwide (3–5), and this global HPAI virus dissemination is caused by migratory wild birds (6). The HPAI crisis appears to be a great threat to not only animal health but also public health worldwide. Furthermore, the World Health Organization reported 860 human infection cases of avian influenza A subtype H5N1 (7) after the first human case of HPAI subtype H5N1, which was reported in Hong Kong in 1997 (8).

In South Korea, outbreaks of three different subtypes of HPAI occurred between 2003 and 2018. The first outbreak of H5N1 occurred from December 2003 to February 2004 and had a high mortality rate at poultry farms, especially among chickens (9). Since then, outbreaks of H5N1 have occurred in 2006, 2008, and 2010 (10–12). The novel HPAI subtype, H5N8, was first reported in January 2014 at South Korean poultry farms (13). Genetic analyses of viruses isolated from wild birds and poultry farms showed that migratory birds could be responsible for the first wave of H5N8 outbreaks between January and May 2014 (14). After the first wave, two waves of subtype H5N8 occurred during September 2014 to June 2015 and during September 2015 to November 2015 (15). It was reported that these sporadic outbreaks were caused by viruses reintroduced into Korea by migratory waterfowl (16). In November 2016, a novel genotype of H5N6 that was first detected in wild birds in Korea and HPAI infectious cases was reported at poultry farms (17). Another novel H5N8 virus co-circulated with H5N6 virus during the outbreaks in 2016, from February to June 2017 (18). In November 2017, the novel H5N6 virus was detected at a broiler duck farm and in wild mallards, with infection spreading to poultry farms (19).

The main strategies used to prevent and control HPAI outbreaks are based on the prohibition of movement, preemptive culling, and vaccinations in infected areas (20). Therefore, it is important to understand pathogen transmissibility in a population to establish an effective disease prevention policy. The basic reproduction number (*R*_0_) is one of the important epidemiologic parameters necessary to understand the characterization of disease and the dynamics in a population (21). *R*_0_is generally defined as the average number of secondary cases caused by one infectious individual during the entire infectious period in an uninfected population (22). If each infected individual infects more than one other individual, on an average, at any time point, then the epidemic will be sustainable (23). Various methods are used to estimate the reproduction number (24–26), and these have been implemented in the R program (27) and Excel (28) as ready-made procedures.

Reproduction number estimation has been used to understand HPAI epidemic characteristics and to provide insight regarding control measures for epidemics. These farm-to-farm reproduction number estimations were targeted to the HPAI subtype H5N1 and were conducted in Nigeria (29), Romania (30), Thailand (31), Bangladesh (32), India (33), Italy, Canada, and the Netherlands (34). In Korea, there was a mathematical modeling study of the reproduction number for HPAI from 2016 to 2017, but this was limited to the local reproduction number and did not include all epidemics from South Korea (35). We aimed to estimate the serial interval and *R*_0_ of HPAI subtypes H5N1, H5N8, and H5N6, which were associated with nine outbreaks from 2003 to 2018 in Korea, and demonstrate the characterization of each subtype by analyzing HPAI characteristics, including the epidemic days, number of farms, species distribution, serial interval, and *R*_0_. It is expected that the results of the present study will become a foundation for demonstrating the disease dynamics of each HPAI subtype and its characteristics, as well as for establishing effective HPAI control, not only for traditional HPAI subtype H5N1 but also the emerging subtypes H5N8 and H5N6.

## Materials and Methods

### Data Collection

**Figure 1:**
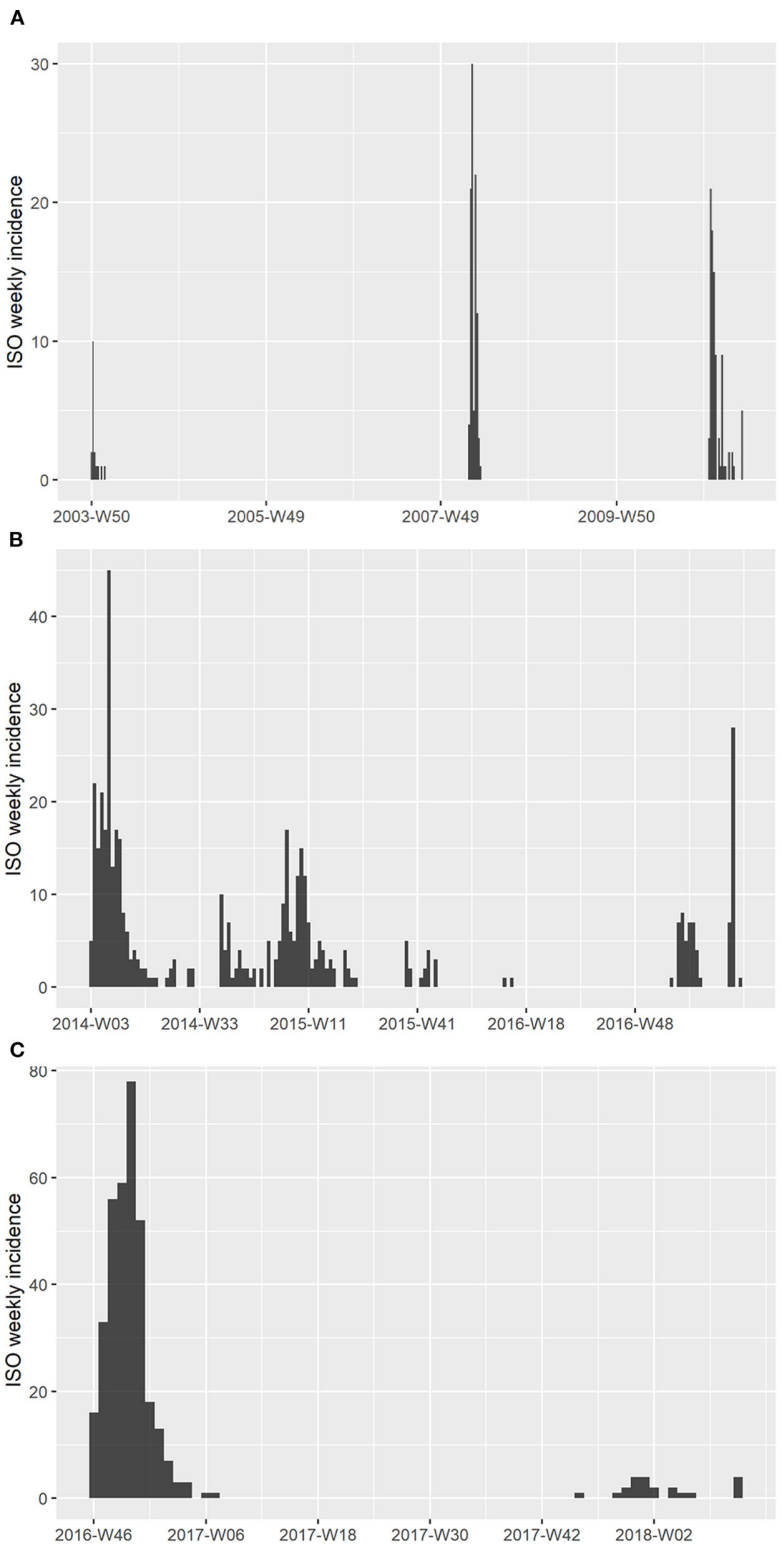
Epidemic curve of HPAI outbreaks in Korea between 2003 and 2018. **(A)** Weekly epidemic case number of HPAI subtype H5N1 from 2003 to 2011. **(B)** Weekly epidemic case number of HPAI subtype H5N8 from 2014 to 2017. **(C)** Weekly epidemic case number of HPAI subtype H5N6 from 2016 to 2018. The x-axis represents the week numbers, which were based on the ISO 8601 week date system.

The epidemic data of HPAI outbreaks in Korea were collected by the Animal and Plant Quarantine Agency (APQA) in Gimcheon, Korea (Table 1). In Korea, three HPAI subtypes occurred from 2003 to 2017, HPAI subtype H5N1 occurred in a total of 214 poultry farms, H5N8 occurred in 469 farms, and H5N6 occurred in total 362 farms. The livestock owner (including the manager) or veterinarian who found an animal with clinical signs and suspected HPAI was required to report the case to the APQA according to the Prevention of Contagious Animal Disease Act. Cloacal, fecal, and blood samples were collected from sick or dead poultry in reported poultry farms, and HPAI virus was confirmed using reverse-transcriptase polymerase chain reaction at the Avian Influenza Research and Diagnosis Department of the APQA. If the suspected farm was confirmed as HPAI-positive and deemed an infected premise (IP), then depopulation of farms with infected poultry and depopulation of all poultry farms in the protection zone were conducted. If a depopulated farm was found to be positive, then it was defined as a positive premise (PP) (36). Both IP and PP were considered cases in this study. The epidemic curve of these HPAI cases was depicted using the “incidence” package in R (37) to illustrate the weekly reported number of poultry farms in the International Organization for Standardization (ISO) week date system (37) (Figure 1). In Korea, there were no poultry farms infected with two HPAI subtypes simultaneously, and each farm only had one subtype in each outbreak.

**Table 1:** HPAI epidemic in Korea from 2003 to 2018.

**Subtype**	**Year of**	**Clade**	**Date**	**Days of**	**Total number**	**Cases**	**No. of chicken**	**No. of duck**	**No. of other**
	**epidemic**			**epidemic**	**of Farms**	**per day**	**farms (%)**	**farms (%)**	**poultry farms (%)**
H5N1	2003	2.5	10/12/2003–05/02/2004	58	18	0.310	7 (38.9)	11 (61.1)	0 (0.0)
	2006	2.2	25/11/2006–06/03/2007	103	7	0.068	4 (57.1)	2 (28.6)	1 (14.3)
	2008	2.3.2	01/04/2008–24/05/2008	54	98	1.815	80 (81.6)	17 (17.3)	1 (1.0)
	2010	2.3.2	29/12/2010–23/05/2011	146	91	0.623	38 (41.8)	50 (54.9)	3 (3.3)
H5N8	2014 1st	2.3.4.4	16/01/2014–29/07/2014	194	212	1.093	39 (18.4)	166 (78.3)	7 (3.3)
	2014 2nd	2.3.4.4	24/09/2014–10/06/2015	260	162	0.623	39 (24.1)	117 (72.2)	6 (3.7)
	2014 3rd	2.3.4.4	14/09/2015–15/11/2015	63	17	0.270	0 (0.0)	14 (82.4)	3 (17.6)
	2014 4th	2.3.4.4	23/03/2016–05/04/2016	14	2	0.143	0 (0.0)	2 (100.0)	0 (0.0)
	2016 1st	2.3.4.4	06/02/2017–14/04/2017	58	40	0.690	16 (40.0)	23 (57.5)	1 (2.5)
	2016 2nd	2.3.4.4	02/06/2017–19/06/2017	18	36	2.000	30 (83.3)	0 (0.0)	6 (16.7)
H5N6	2016	2.3.4.4	16/11/2016–18/02/2017	95	340	3.579	192 (56.5)	140 (41.2)	8 (2.4)
	2017	2.3.4.4	19/11/2017–18/03/2018	121	22	0.182	8 (36.4)	14 (63.6)	0 (0.0)

Based on the APQA epidemiology reports, the HPAI outbreaks were classified as waves when the period between cases was longer than 1 month (38). As a result of this classification, outbreaks of the subtype H5N8, which occurred in 2014 and 2016, were classified as four and two waves, respectively. Four outbreaks, including the H5N1 outbreak in 2003, outbreak in 2006, the fourth wave of the H5N8 outbreak in 2014, and the second wave of the H5N8 outbreak in 2016, were excluded from the analysis because the samples were too small to calculate *R*_0_.

### Serial Interval and Generation Time

A serial interval is the time between successive cases in a chain of transmission, estimated from the interval between clinical onsets in patients (25). We estimated the serial interval of HPAI as the time between the reported date of the first farm with infected cases and secondary farm with infected cases. This estimation was based on the investigation of the epidemic pathway of HPAI transmission, which shows the epidemiologic relationship between the infector and infectee. According to the APQA investigations, HPAI transmission could be possible through wild migratory birds, wild animals, farm owners, managers, staff, vehicles related to the poultry industry, and airborne transmission from nearby infected farms. The epidemic transmission pathway investigation was conducted by an APQA epidemiologic investigator visiting and interviewing the places suspected to be associated with the infected farms, including animal facilities such as hatcheries, feed factories, and live bird markets. The APQA investigated vehicles, people, livestock, and their products that entered an infected farm from 21 days prior to infection and estimated the disease transmissions.

In addition to investigating via interview, the APQA used geographic information to identify HPAI viral transmission by vehicles. In Korea, vehicles related to the poultry industry transporting poultry, poultry products, medicines, feed, and feces must be registered with the Korea Animal Health Integrated System (KAHIS; http://www.kahis.go.kr). The movements of livestock-related vehicles are reported to the KAHIS, making it possible to track the movement of vehicles, people, livestock, and animal products.

Through these interviews and vehicle information, the disease transmission pathway via transportation and human movement was identified. If a clear epidemiologic link to the infected farm could not be found through interviews and movement tracking, then we hypothesized that the farm might have been infected with HPAI by wild migratory birds or wild animals. We then excluded infection thought to be caused by wild birds or wild animals during the estimation of the serial interval because it is not possible to observe the serial interval of virus transmission from wild birds and animals.

**Figure 2:**
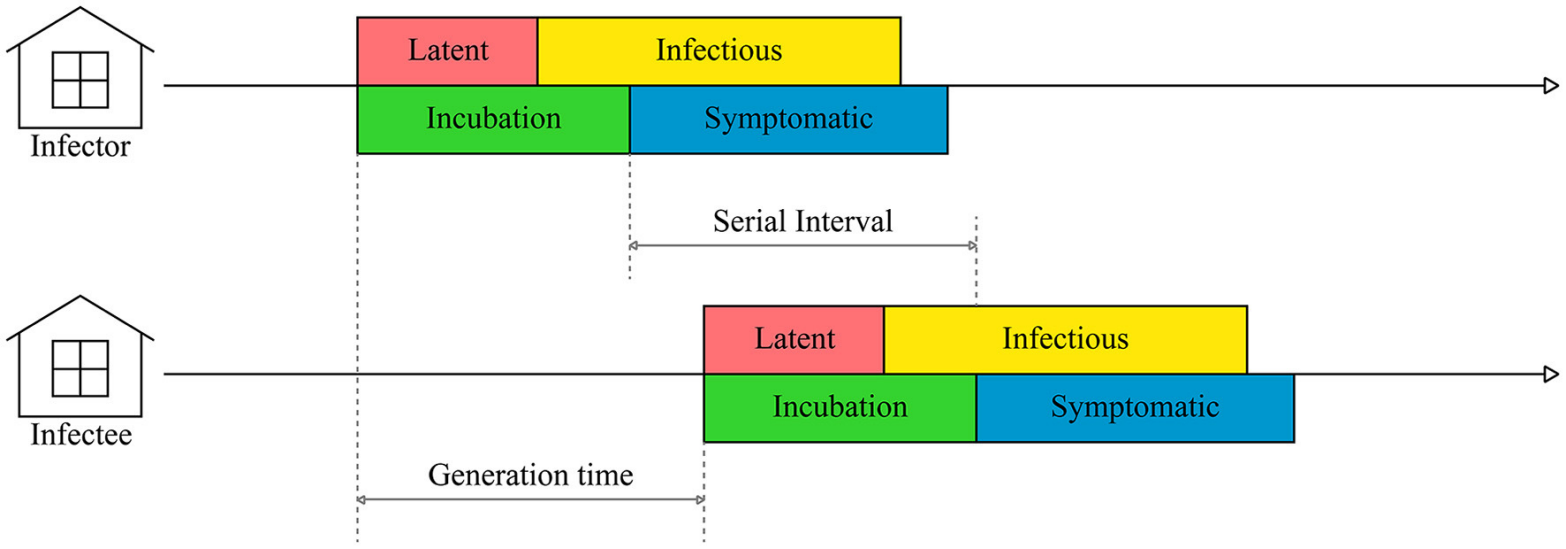
A schematic representation of HPAI transmission between farms.

The generation time is the modeling term describing the time duration from the onset of transmittable infection in a primary case to the onset of infection in a secondary case infected from the primary case. We defined the generation time as the difference between suspected infection days of the primary farm and secondary farm, which was measured through epidemiologic investigation (Figure 2). The suspected infection day was estimated according to the day reported by the farm owner after clinical symptoms were found in the poultry and the period between the infection and latent period of each HPAI subtype in the poultry species. We estimated the suspected infection date from the day the clinical symptoms were reported by subtracting the periods between infection and clinical symptoms. For H5N1, the periods between infection and clinical symptoms were assumed to be 2 days for chickens (9), 4 days for ducks (39), and 3.8 days for other poultry species (9). For H5N8, the periods were 3.2 days for chickens (40), 8.0 days for ducks (15), and 2.0 days for other species (41). For H5N6, the periods were 2.6 days for chickens (42), 4.6 days for ducks (43), and 3.0 days for other species (43).

**Figure 3:**
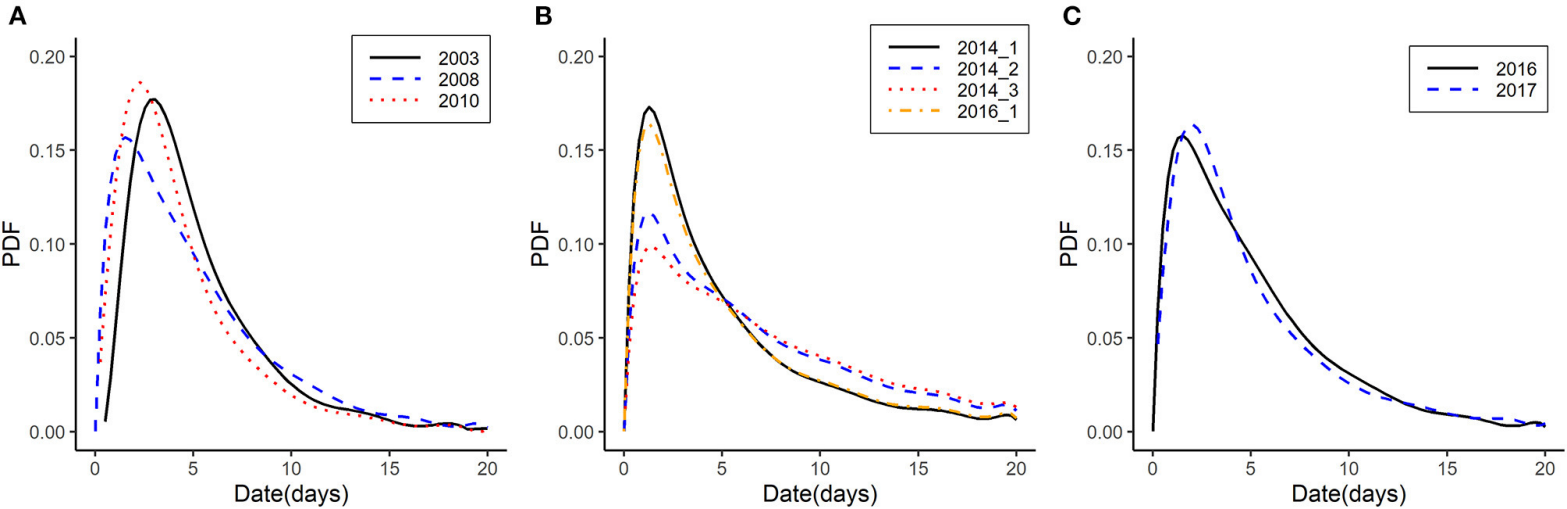
Generation time distribution of HPAI outbreaks from 2003 to 2018 in Korea by HPAI subtype. **(A)** Generation time distribution of HPAI subtype H5N1 in 2003, 2008, and 2010. **(B)** Generation time distribution of HPAI subtype H5N8 during the 2014 first wave, second wave, and third wave and during the 2016 first wave. **(C)** Generation time distribution of HPAI subtype H5N6 in 2016 and 2017. The x-axis represents the days for generation time. The y-axis represents the probability density function (PDF).

Based on the generation time between case farms, we calculated the discretized generation time distribution using a function (est.GT) in the R0 package (27). Discretization is performed on the grid [0, 0.5), [0.5, 1.5), [1.5, 2.5), etc… where the unit is time interval of days (27). Time-to-event data were assumed to follow a parametric distribution with a probability density function (PDF). The distribution of generation time is expressed in the form of parametric distribution such as “gamma,” “lognormal,” or “Weibull,” using maximum likelihood. The mean and standard deviation of generation time is provided in the desired time units. The calculated distribution of the generation time in each subtype and outbreaks is depicted in Figure 3.

### Model Assumption and Data Analysis

The study model is based on the susceptible-infected-removed (SIR) compartmental model (44), which divides poultry farms into compartment. A susceptible farm (S) becomes infectious (I) through contact with the possible disease and is then removed (R) by depopulation. The dynamics of an epidemic can be described as the equation given below when *N* is the sum of *S, I*, and *R*.

$$\begin{array}{ll} \frac{dS}{dt} & =-\frac{\beta IS}{N}\\ \frac{dI}{dt} & =\frac{\beta IS}{N}-\gamma I\\ \frac{dR}{dt} & =\gamma I \end{array}$$

In this model, β is a parameter, which controls how much the disease can be transmitted through the exposure of HPAI virus, and γ is a parameter, which expresses how many poultry farms can be removed in a specific period. In this model, the average number of secondary infections caused by an infected host, *R*_0_, equals β/ γ (45).

We constructed exponential growth (EG) and maximum likelihood (ML) models to estimate early reproduction numbers using the R0 package (27) in R (version 3.3.0). The EG model assumes that the initial reproduction ratio can be associated with the EG rate during the early epidemic phase (24). The formula is *R*_0_ = 1/M (–*r*), where *r* denotes the initial EG rate and M stands for the moment generating function of generation time distribution. In the initial EG model, a period from day 1 to day 14 of the epidemics was chosen when the outbreak's growth was exponential. The 14-day interval was selected based on Korea's standstill policy (38). When HPAI outbreaks are reported in South Korea, a standstill policy is implemented for the vehicles, to reduce the spread of HPAI. This policy is intended to minimize the contact between vehicles and suppress the HPAI dissemination. We determined that this intervention affects the basic reproduction number of HPAI. Therefore, we specified the exponential growth for the first 14 days of the epidemic wave in each case.

A function (est.R0.EG) in the R0 package was used (27). We used a sensitivity test in EG to select the period during which growth is exponential as optimized time windows. We used the “sensitivity analysis” function to compute the deviance R-squared statistic over a range of periods.

The ML estimation model assumes that the number of secondary cases caused by an index case is Poisson-distributed with the expected value *R*_0_ (25). The log-likelihood (LL) of *R*_0_ was defined as $LL\left(R_{0}\right)=\overset{T}{\underset{t=1}{\sum }}\log\left(\frac{e^{-\mu_{t}}\mu_{t}^{N_{t}}}{N_{t}!}\right)$, where $\mu_{t}=R_{0}\overset{t}{\underset{i=1}{\sum }}N_{t-i}w_{i}$. This model assume that the number of new cases at indexing time *t* as N= {*N*_*t*_}, *t* = 0,…T, a generation time distribution *w*, and μ_*t*_ which represent the total number of cases produced by the earlier case *N*_*t*_. The likelihood must be calculated on a period of exponential, and the deviance R-squared measure may be used to select the best period that maximized the likelihood. In this study, the range was set as 0.01–50, in which the maximum must be searched. A function (est.R0.ML) in the R0 package was used (27). The goodness of fit of each model was calculated using the chi-square goodness of fit test in R.

The Kruskal-Wallis test was used to determine the statistical differences in epidemics between subtypes (46). The epidemic days, number of farms, cases per day, poultry species distribution of farms, generation time, and *R*_0_ estimated by EG and ML of the three subtypes H5N1, H5N8, and H5N6 were analyzed. The significance level was α = 0.05. These statistical analyses were performed using SPSS 22.0 (IBM, Armonk, NY, USA).

## Results

### HPAI Epidemic in Korea

We investigated 12 HPAI outbreaks of three subtypes, H5N1, H5N8, and H5N6, that occurred from 2003 to 2018 in Korea. Table 1 presents a summary of the epidemic data, including the period of outbreaks and the number of infected farms that were investigated. The weekly epidemic curves of HPAI outbreaks are shown in Figure 1 based on the ISO 8601 week date system. The H5N1 HPAI outbreaks (except for the 2008 outbreaks) began between November and February, when the lowest temperature drops below 0°C (Figure 1A). Regarding H5N8 in 2014, the second and third waves recurred in September 2015 and 2016, respectively (Figure 1B). However, the second wave of H5N6 in 2016 occurred in June (Figure 1C). The longest outbreak was the second wave of H5N8 in 2014, which occurred over 260 days. The shortest outbreak was the fourth wave of H5N8 in 2014, which occurred over 14 days. The outbreaks with the most cases (340 poultry farms) and cases per day (3.579 cases per day) were the H5N6 outbreaks in 2016. Regarding H5N8 in 2014, more than 72% of the occurrences were in ducks; however, there was no apparent species specificity for subtypes H5N1 and H5N6.

### Serial Interval and Basic Reproduction Number of HPAI in Korea

We selected nine outbreaks with sufficient number of premises to calculate *R*_0_ and analyzed the generation time and initial *R*_0_ using the EG and ML methods (Table 2). Generation time distributions are illustrated by each HPAI subtype as the PDF in Figure 3. Generation time of H5N1 were estimated between 4.58 and 5.24 days (Figure 3A), generation time of H5N8 were estimated to have 6 days or more (6.01–8.23 days) (Figure 3B), and generation time of H5N6 were estimated between 5.02 and 5.91 days (Figure 3C). *R*_0_ was estimated as 1.65–2.20 for subtype H5N1, 0.03-1.56 for subtype H5N8, and 1.03–1.24 for subtype H5N6 using EG methods. Using ML methods, *R*_0_was estimated as 1.68–1.95 for subtype H5N1, 1.03–1.83 for subtype H5N8, and 1.37–1.60 for subtype H5N6.

**Table 2:** Generation time and reproduction number of HPAI by EG and ML method.

**Subtype**	**Year of epidemic**	**Distribution**	**Mean generation time (95% CI) (Days)**	**Initial *R_**0**_* by EG Method (95% CI)**	****χ**^2^ of EG Method**	**Initial *R_**0**_* by ML Method (95% CI)**	****χ**^2^ of ML Method**	**Optimal time windows (percent in total period)**	***R_**0**_* by EG Method (optimal) (95% CI)**
H5N1	2003	Lognormal	5.24 (3.51–6.97)	2.02 (1.02–3.76)	0.33	1.95 (0.81–3.86)	0.33	9–46 (65.52%)	0.18 (0.01–0.51)
	2008	Gamma	4.98 (4.15–5.81)	1.65 (1.02–2.49)	0.33	1.68 (0.92–2.76)	0.34	9–54 (85.19%)	0.74 (0.65–0.82)
	2010	Gamma	4.58 (3.76–5.40)	2.20 (1.51–3.16)	0.30	1.93 (1.10–3.10)	0.30	9–138 (89.04%)	0.77 (0.72–0.83)
H5N8	2014 1st	Lognormal	7.45 (5.83–9.07)	1.56 (0.95–2.23)	0.31	1.83 (1.11–2.81)	0.31	14–125 (57.73%)	0.72 (0.65–0.79)
	2014 2nd	Weibull	8.23 (6.94–9.52)	0.35 (0.00–1.38)	0.36	1.56 (0.70–2.97)	0.35	10–248 (91.92%)	1.01 (0.99–1.03)
	2014 3rd	Weibull	7.39 (4.39–10.39)	0.03 (0.00–0.98)	0.36	1.03 (0.22–2.88)	0.38	10–50 (65.08%)	2.17 (1.26–3.67)
	2016 1st	Weibull	6.01 (4.57–7.45)	1.23 (0.50–2.31)	0.34	1.70 (0.75–3.22)	0.37	2–45 (75.86%)	1.37 (1.13–1.16)
H5N6	2016	Gamma	5.02 (4.56–5.48)	1.24 (0.87–1.73)	0.36	1.60 (1.09–2.25)	0.36	14–94 (85.26%)	0.71 (0.67–0.74)
	2017	Lognormal	5.91 (3.14–8.68)	1.03 (0.01–2.45)	0.38	1.37 (0.34–3.56)	0.38	14–107 (77.69%)	0.90 (0.78–1.01)

*SD, standard deviation; EG, exponential growth; ML, maximum likelihood estimation; CI, confidence interval*.

Most of the *R*_0_in the EG and ML methods were similar, except for the second and third waves of H5N8 in 2014. The R value obtained by the EG method was <1 for the second and third waves of H5N8 in 2014. To select the optimal time windows, sensitivity results of the time windows and *R*_0_ were used (Table 2). Optimized time windows selected by sensitivity tests accounted for 69.14% of the outbreak periods, on an average, and the optimal *R*_0_ values in optimized time windows were <1 for the subtypes H5N1 and H5N6 outbreaks.

### Epidemic Statistics Between Subtypes

The average values of the number of epidemic days, infected poultry farms, species distribution, and infected farms per day for the three subtypes of nine selected outbreaks were determined (Table 3). The average numbers of epidemic days were 86.0 for H5N1, 108.0 for H5N6, and 143.8 for H5N8. The average numbers of farms were 69.0 for H5N1, 107.8 for H5N8, and 181.0 for H5N6. Regarding the species distribution, subtype H5N8 was more highly distributed among duck farms (74.2%) than other subtypes (37.7% for H5N1 and 42.5% for H5N6).

**Table 3:** Epidemic characteristics, mean generation time, and *R*_0_ in two models by HPAI subtype H5N1, H5N8, and H5N6.

**Subtype**	**Average**	**Average**	**Cases**	**Chicken (%)**	**Duck (%)**	**Etc. (%)**	**Mean generation**	***R_**0**_* by EG Method**	***R_**0**_* by ML Method**
	**epidemic days**	**number of farms**	**per day**				**time (days)**	**(95% CI)**	**(95% CI)**
H5N1	86.0	69.0	0.802	41.7 (60.4)	26.0 (37.7)	2.0 (2.9)	4.80 (4.23–5.38)	1.96 (1.48–2.39	1.69 (1.10–2.28)
H5N8	143.8	107.8	0.750	23.5 (21.8)	80.0 (74.2)	4.3 (3.9)	7.58^*^(6.63–8.46)	1.49 (1.19–1.79)	1.60 (0.97–2.23)
H5N6	108.0	181.0	1.676	100.0 (55.2)	77.0 (42.5)	4.0 (2.2)	5.09 (4.44–5.74)	1.14 (0.76–1.51)	1.49 (0.94–2.04)

*EG, exponential growth; ML, maximum likelihood estimation; CI, confidence interval*.

* *Mean generation time of subtype H5N8 is significantly longer than subtype H5N1 (P = 0.03)*.

The Kruskal-Wallis H test showed a statistically significant difference in mean generation time among the different subtypes [χ^2^ (2) = 6.444; *p* = 0.040], with mean rank scores of 2.33 for subtype H5N1, 7.50 for H5N8, and 4.00 for H5N6. The pairwise comparison showed that the mean H5N8 generation time (7.58 days) was significantly longer than the H5N1 generation time (4.80 days) (*P* = 0.03) (Table 3). There were no significant differences among subtypes in epidemic days, number of farms, cases per day, species distributions, or reproduction number.

## Discussion

HPAI outbreaks occur continually worldwide and have become a major threat to animal and human public health. In South Korea, eight outbreaks with multiple waves of infections occurred between 2003 and 2018; these involved three different HPAI subtypes, H5N1, H5N8, and H5N6, and massively damaged the poultry industry. Therefore, it is important to understand the HPAI transmissibility at poultry farms to control outbreaks by establishing an effective prevention policy. An effective tool for understanding disease characteristics is the *R*_0_, which is generally defined as the average number of secondary cases caused by one infected individual (21). Therefore, we investigated the transmission dynamics of the HPAI subtypes H5N1, H5N8, and H5N6 by estimating the generation time and *R*_0_. To the best of our knowledge, no previous study has attempted to estimate *R*_0_ of various HPAI subtypes and perform comparative analyses among them. This could be the first study to investigate the disease transmission dynamics of HPAI subtypes H5N1, H5N8, and H5N6, which are emerging worldwide.

The *R*_0_ of HPAI H5N1 in Korea estimated in this study was between 1.68 and 1.95, according to the ML method (Table 1). The *R*_0_ of subtype H5N1 has previously been estimated in countries such as Italy (1.2–2.7), Canada (1.4–2.7), the Netherlands (1.0–3.0) (34), Romania (1.95–2.68) (30), Bangladesh (0.85–0.96) (32), and Thailand (1.27–1.60) (47). Despite being the same subtype of HPAI, the estimated *R*_0_ subtype H5N1 varied across countries. We assumed that several factors, such as geographic distribution of poultry farms, mixed farming systems, poultry product supply system, and climate, were associated with this difference.

We believe that unique characteristics of the poultry industry in Korea and climatic differences are the major causes for these observed differences. We speculate that the estimated *R0* may be related to characteristics of the Korean poultry industry, such as the coexistence of large-scale commercial farms and small family farms. Among the Organization for Economic Cooperation and Development (OECD) countries, Korea has the lowest availability of arable land per capita (0.03 hectare in 2016) (48). This land scarcity is an important factor leading to high stocking densities (49). A previous study suggested that farms with large flocks and the presence of a neighboring farm within 500 m were risk factors of HPAI at Korean broiler duck farms (50). This high stocking and local density of large-scale poultry farms could increase the likelihood of massive infections when HPAI outbreaks occur in Korea.

Small family poultry farms also represent a biosecurity risk during HPAI outbreaks. Most of these small farms sell live poultry to local markets without going through slaughterhouses; this could be a pathway for the spread of HPAI viruses. Additionally, there was an obvious lack of information regarding the official statistics of poultry farms too small to be defined as agricultural holders in Korea (51). This includes establishments with <0.1 hectares of land or with sales of agricultural products per year or value of agricultural animals less than KRW 1.2 million (USD 1,090).

Secondly, we hypothesize that climate factors during the epidemic period may affect *R*_0_ in these countries. Climate factors could affect HPAI transmission and persistence by altering bird migration, virus shedding between hosts, and virus survival outside the host (52). Climate change is considered to influence the wild bird species composition and their migration cycle, and these changes will affect the transmission intensity of disease (53). Furthermore, temperature and humidity could be related to viral persistence in the host and environment. An influenza virus transmission experiment using a guinea pig model suggested that relative low humidity and cold temperature were favorable for spreading influenza (54). Liu et al. (55) showed that the environmental temperature decreased shortly before HPAI H5N1 outbreaks in domestic poultry in Eurasia between 2005 and 2006. Additionally, AI viral infectivity remained at lower temperatures (<17°C) during an *in vivo* test (56). Therefore, it is assumed that our estimated *R*_0_ in Korea is higher than the *R*_0_ in Thailand and Bangladesh, where the average annual temperatures and humidity are higher. Based on these results, we assumed that the climate factors were closely related to the *R*_0_estimated in several countries in terms of virus transmission and survivability.

In 2016, two novel HPAI subtypes, H5N6 and H5N8, occurred simultaneously. HPAI H5N6 occurred from November 2016 to February 2017, whereas subtype H5N8 occurred from February to April 2016; the first wave and second wave occurred in June. Although these two subtypes occurred simultaneously, both were novel viruses. The genetic clade analysis suggested that Korean H5N6 viruses are novel reassortments of multiple virus subtypes, and it is difficult for H5N6 virus reassortment to occur during outbreaks that could increase the possibility of viral subtype mutation (5). Additionally, an infection experiment involving wild mandarin ducks demonstrated a difference in viral shedding and viral tropism in H5N8 and H5N6 viruses within the same clade of 2.3.4.4 H5 HPAI viruses (57). Based on these findings, both subtypes were independent of each other, and the virus infectivity could also be different; therefore, different *R*_0_ was expected.

However, our estimated initial *R*_0_ value in 2016 suggested a similarity between the reproduction number represented in subtypes H5N8 (1.70) and H5N6 (1.60) (Table 2). Apart from the difference in transmissibility of each virus subtype, the level of transmission between farms in the field may be similar between the two subtypes. However, this presumes that the values of *R*_0_ of the two subtypes were similarly calculated because the biosecurity policy implemented during the outbreaks was identical. The basic reproductive number is affected by the rate of contacts in the host population, the probability of infection being transmitted during contact, and the duration of infectiousness (58). Therefore, it can be estimated that the *R*_0_ of two different subtypes were similar due to the reduction of the poultry population through preemptive culling and the reduction of contact between farms because of the standstill (59).

The quarantine against HPAI in Korea has changed over 14 years after the first HPAI epidemic in 2003. The HPAI prevention policy changed dramatically, especially before and after H5N8 epidemics in 2014. Before the outbreaks, Korea Animal Health Integrated System (KAHIS) was established in 2013 to monitor livestock vehicle movement. In this system, all poultry-related vehicles must be registered with KAHIS and equipped with a global positioning system mandatorily (60). Also during the epidemics, the preemptive depopulation of the protective zone was changed from a radius of 500 m−3 km, and inspections were conducted more than once before releasing poultry and poultry products (36), The influence of these quarantine policy can also be seen in the changes in the *R*_0_ values of each wave of subtype H5N8 that occurred between 2014 and 2016. For H5N8 in 2014, the initial *R*_0_ of each wave showed a tendency to decrease as the outbreak progressed gradually (Table 2). This would indicate that the effectiveness of control measures for HPAI were increasing while the waves were passing.

In the Kruskal-Wallis model, H5N1 and H5N8 subtypes showed statistically significant differences in generation time (*P* = 0.03) (Table 3). However, there were no significant differences in the epidemic characteristics of the subtypes. There was also no statistical significance in the *R*_0_ obtained through the EG and ML models. This generation time difference in the two subtypes might be associated with subtype pathogenicity in the poultry species. The spread of H5N1 viruses in the field was quickly controlled as a result of the rapid diagnosis of the infections due to the high pathogenicity of these viruses in poultry. In contrast, subtypes H5N6 and H5N8 clustered as clade 2.3.4. H5NX viruses are usually mild in ducks, leading to delayed diagnosis of infections and persistent spread in the wild (61). Therefore, the H5N8 subtype could possibly spread the HPAI virus over a relatively longer period than the H5N1 subtype which could be driven by sub-clinical spread in ducks.

In conclusion, this study showed the characterization of each subtype by analyzing the HPAI characteristics, including the epidemics, number of farms, species distribution, generation time, and *R*_0_ of HPAI subtypes H5N1, H5N8, and H5N6, which were associated with nine outbreaks in Korea between 2003 and 2018. *R*_0_, which is estimated by the generation time, index case, and secondary cases, is essential for identifying the characteristics of HPAI. In particular, our findings suggest that the estimated *R*_0_ might be influenced by the HPAI subtype and might be associated with the seasonal aspects during the early stage, species specificity by virus subtype, and prevention policy. We believe that the results of the present study are helpful for demonstrating the disease dynamics of each HPAI subtype and its characteristics and, thus greatly assist in better disease control strategies. It could be possible to establish systematic quarantine policies to reduce the socio-economic losses caused by HPAI, Especially differences observed between countries with different poultry raising systems and climatic conditions. This study provided insight regarding HPAI transmission of the traditional subtype H5N1 and newly emerging subtypes H5N8 and H5N6. Further research on the basic reproduction numbers of the HPAI subtypes occurring worldwide is required to understand the global dynamics of HPAI transmission.

## Data Availability Statement

The original contributions presented in the study are included in the article/supplementary material, further inquiries can be directed to the corresponding author/s.

## Author Contributions

W-HK designed the study, investigated data collection, reviewed the data, performed data analysis, and participated in manuscript preparation. SC supervised the project, administrated the project, acquired funds, and participated in the manuscript review.

## Conflict of Interest

The authors declare that the research was conducted in the absence of any commercial or financial relationships that could be construed as a potential conflict of interest.

## References

[1] Alexander DJ. An overview of the epidemiology of avian influenza. Vaccine. (2007) 25:5637–44. 10.1016/j.vaccine.2006.10.051 17126960

[2] Short KR Richard M Verhagen JH van Riel D Schrauwen EJ van den Brand JM. One health, multiple challenges: the inter-species transmission of influenza A virus. One Health. (2015) 1:1–13. 10.1016/j.onehlt.2015.03.001 26309905 PMC4542011

[3] Gu M Zhao G Zhao K Zhong L Huang J Wan H. Novel variants of clade 2.3. 4 highly pathogenic avian influenza A (H5N1) viruses, China. Emerg Infect Dis. (2013) 19:2021. 10.3201/eid1912.130340 24274396 PMC3840869

[4] DeJesus E Costa-Hurtado M Smith D Lee D-H Spackman E Kapczynski DR. Changes in adaptation of H5N2 highly pathogenic avian influenza H5 clade 2.3. 4.4 viruses in chickens and mallards. Virology. (2016) 499:52–64. 10.1016/j.virol.2016.08.036 27632565 PMC5102764

[5] Si Y-J Lee IW Kim E-H Kim Y-I Kwon H-I Park S-J. Genetic characterisation of novel, highly pathogenic avian influenza (HPAI) H5N6 viruses isolated in birds, South Korea, November 2016. Euro Surveill. (2017) 22:30434. 10.2807/1560-7917.ES.2017.22.1.30434 28079520 PMC5388099

[6] Verhagen JH Herfst S Fouchier RA. How a virus travels the world. Science. (2015) 347:616–7. 10.1126/science.aaa6724 25657235

[7] World Health Organization. Cumulative Number of Confirmed Human Cases for Avian Influenza A(H5N1) Reported to WHO, 2003-2019. (2019). Available online at: https://www.who.int/ (accessed 24 May, 2019).

[8] De Jong J Claas E Osterhaus A Webster R Lim W. A pandemic warning? Nature. (1997) 389:554. 10.1038/39218 9335492 PMC7095477

[9] Lee C-W Suarez DL Tumpey TM Sung H-W Kwon Y-K Lee Y-J. Characterization of highly pathogenic H5N1 avian influenza A viruses isolated from South Korea. J Virol. (2005) 79:3692–702. 10.1128/JVI.79.6.3692-3702.2005 15731263 PMC1075707

[10] Kim H-R Park C-K Lee Y-J Woo G-H Lee K-K Oem J-K. An outbreak of highly pathogenic H5N1 avian influenza in Korea, 2008. Vet Microbiol. (2010) 141:362–6. 10.1016/j.vetmic.2009.09.011 19800184

[11] Lee Y-J Choi Y-K Kim Y-J Song M-S Jeong O-M Lee E-K. Highly pathogenic avian influenza virus (H5N1) in domestic poultry and relationship with migratory birds, South Korea. Emerg Infect Dis. (2008) 14:487. 10.3201/eid1403.070767 18325269 PMC2570817

[12] Kim H-R Lee Y-J Park C-K Oem J-K Lee O-S Kang H-M. Highly pathogenic avian influenza (H5N1) outbreaks in wild birds and poultry, South Korea. Emerg Infect Dis. (2012) 18:480. 10.3201/1803.111490 22377052 PMC3309593

[13] Lee Y. Novel Reassortant Influenza A (H5N8) Viruses, South Korea, 2014. Emerg Infect Dis. J. (2014) 20:1087–9. 10.3201/eid2006.140233 24856098 PMC4036756

[14] Jeong J Kang H-M Lee E-K Song B-M Kwon Y-K Kim H-R. Highly pathogenic avian influenza virus (H5N8) in domestic poultry and its relationship with migratory birds in South Korea during 2014. Vet Microbiol. (2014) 173:249–57. 10.1016/j.vetmic.2014.08.002 25192767

[15] Animal and Plant Quarantine Agency. 2014-2016 Epidemiologic Reports of Highly Pathogenic Avian Influenza. Animal and Plant Quarantine Agency (2016) .

[16] Kwon J-H Lee D-H Swayne DE Noh J-Y Yuk S-S Erdene-Ochir T-O. Highly pathogenic avian influenza A (H5N8) viruses reintroduced into South Korea by migratory waterfowl, 2014–2015. Emerg Infect Dis. (2016) 22:507. 10.3201/eid2203.151006 26890406 PMC4766904

[17] Lee E-K Song B-M Lee Y-N Heo G-B Bae Y-C Joh S-J. Multiple novel H5N6 highly pathogenic avian influenza viruses, South Korea, 2016. Infect Genet Evol. (2017) 51:21–3. 10.1016/j.meegid.2017.03.005 28284997

[18] Kim Y-I Park S-J Kwon H-I Kim E-H Si Y-J Jeong J-H. Genetic and phylogenetic characterizations of a novel genotype of highly pathogenic avian influenza (HPAI) H5N8 viruses in 2016/2017 in South Korea. Infect Genet Evol. (2017) 53:56–67. 10.1016/j.meegid.2017.05.001 28477974

[19] Lee E-K Lee Y-N Kye S-J Lewis NS Brown IH Sagong M. Characterization of a novel reassortant H5N6 highly pathogenic avian influenza virus clade 2.3. 4.4 in Korea, 2017. Emerg Microbes Infect. (2018) 7:103. 10.1038/s41426-018-0104-3 29895932 PMC5997646

[20] Yee KS Carpenter TE Cardona CJ. Epidemiology of H5N1 avian influenza. Comp Immunol Microbiol Infect Dis. (2009) 32:325–40. 10.1016/j.cimid.2008.01.005 18448168

[21] de Jong MC. Mathematical modelling in veterinary epidemiology: why model building is important. Prev Vet Med. (1995) 25:183–93. 10.1016/0167-5877(95)00538-2

[22] Thomas JC Thomas JC Weber DJ. Epidemiologic Methods for the Study of Infectious Diseases. Oxford: Oxford University Press (2001) p. 64–5.

[23] Dietz K. The estimation of the basic reproduction number for infectious diseases. Stat Methods Med Res. (1993) 2:23–41. 10.1177/096228029300200103 8261248

[24] Wallinga J Lipsitch M. How generation intervals shape the relationship between growth rates and reproductive numbers. Proc R Soc B. (2006) 274:599–604. 10.1098/rspb.2006.3754 17476782 PMC1766383

[25] Forsberg White L Pagano M. A likelihood-based method for real-time estimation of the serial interval and reproductive number of an epidemic. Stat Med. (2008) 27:2999–3016. 10.1002/sim.3136 18058829 PMC3951165

[26] Wallinga J Teunis P. Different epidemic curves for severe acute respiratory syndrome reveal similar impacts of control measures. Am J Epidemiol. (2004) 160:509–16. 10.1093/aje/kwh255 15353409 PMC7110200

[27] Obadia T Haneef R Boëlle P-Y. The R0 package: a toolbox to estimate reproduction numbers for epidemic outbreaks. BMC Med Inform Decis Mak. (2012) 12:147. 10.1186/1472-6947-12-147 23249562 PMC3582628

[28] Cori A Ferguson NM Fraser C Cauchemez S. A new framework and software to estimate time-varying reproduction numbers during epidemics. Am J Epidemiol. (2013) 178:1505–12. 10.1093/aje/kwt133 24043437 PMC3816335

[29] Bett B Henning J Abdu P Okike I Poole J Young J. Transmission rate and reproductive number of the H 5 N 1 highly pathogenic avian influenza virus during the December 2005–July 2008 Epidemic in Nigeria. Transbound Emerg Dis. (2014) 61:60–8. 10.1111/tbed.12003 22925404

[30] Ward M Maftei D Apostu C Suru A. Estimation of the basic reproductive number (R 0) for epidemic, highly pathogenic avian influenza subtype H5N1 spread. Epidemiol Infect. (2009) 137:219–26. 10.1017/S0950268808000885 18559127

[31] Marquetoux N Paul M Wongnarkpet S Poolkhet C Thanapongtharm W Roger F. Estimating spatial and temporal variations of the reproduction number for highly pathogenic avian influenza H5N1 epidemic in Thailand. Prev Vet Med. (2012) 106:143–51. 10.1016/j.prevetmed.2012.01.021 22365379

[32] Ssematimba A Okike I Ahmed G Yamage M Boender G Hagenaars T. Estimating the between-farm transmission rates for highly pathogenic avian influenza subtype H5N1 epidemics in Bangladesh between 2007 and 2013. Transbound Emerg Dis. (2018) 65:e127–34. 10.1111/tbed.12692 28805017

[33] Pandit PS Bunn DA Pande SA Aly SS. Modeling highly pathogenic avian influenza transmission in wild birds and poultry in West Bengal, India. Sci Rep. (2013) 3:1–8. 10.1038/srep02175 23846233 PMC3807259

[34] Garske T Clarke P Ghani AC. The transmissibility of highly pathogenic avian influenza in commercial poultry in industrialised countries. PLoS ONE. (2007) 2:e349. 10.1371/journal.pone.0000349 17406673 PMC1831494

[35] Lee J Ko Y Jung E. Effective control measures considering spatial heterogeneity to mitigate the 2016–2017 avian influenza epidemic in the Republic of Korea. PLoS ONE. (2019) 14:e0218202. 10.1371/journal.pone.0218202 31194835 PMC6564009

[36] Oh S-m. Self-Declaration of the Recovery of Freedom From Highly Pathogenic Avian Influenza in Poultry by Republic of Korea: OIE Delegate for Republic of Korea, Ministry of Agriculture, Food and Rural Affairs. (2018). Available online at: http://www.oie.int (accessed 27 May, 2019).

[37] Kamvar ZN Cai J Pulliam JR Schumacher J Jombart T. Epidemic curves made easy using the R package incidence. F1000Res. (2019) 8:139. 10.12688/f1000research.18002.1 31119031 PMC6509961

[38] Animal and Plant Quarantine Agency. High Pathogenic Avian Influenza; The Blue Book. Yong-Sang K, editor. Noida, IN: Imun Company (2015).

[39] Jeong O-M Kim M-C Kim M-J Kang H-M Kim H-R Kim Y-J. Experimental infection of chickens, ducks and quails with the highly pathogenic H5N1 avian influenza virus. J Vet Sci. (2009) 10:53–60. 10.4142/jvs.2009.10.1.53 19255524 PMC2801098

[40] Lee E-K Song B-M Kang H-M Woo S-H Heo G-B Jung SC. Experimental infection of SPF and Korean native chickens with highly pathogenic avian influenza virus (H5N8). Poult Sci. (2016) 95:1015–9. 10.3382/ps/pew028 26933235

[41] Lee D-H Kwon J-H Noh J-Y Park J-K Yuk S-S Erdene-Ochir T-O. Pathogenicity of the Korean H5N8 highly pathogenic avian influenza virus in commercial domestic poultry species. Avian Pathol. (2016) 45:208–11. 10.1080/03079457.2016.1142502 26814367

[42] Park S-C Song B-M Lee Y-N Lee E-K Heo G-B Kye S-J. Pathogenicity of clade 2.3. 4.4 H5N6 highly pathogenic avian influenza virus in three chicken breeds from South Korea in 2016/2017. J Vet Sci. (2019) 20:e27. 10.4142/jvs.2019.20.e27 31161745 PMC6538517

[43] Animal and Plant Quarantine Agency. 2016-2017 Epidemiologic Reports of Highly Pathogenic Avian Influenza. Animal and Plant Quarantine Agency (2017).

[44] Iwami S Takeuchi Y Liu X. Avian–human influenza epidemic model. Math Biosci. (2007) 207:1–25. 10.1016/j.mbs.2006.08.001 17010999

[45] Ridenhour B Kowalik JM Shay DK. Unraveling r 0: Considerations for public health applications. Am J Public Health. (2018) 108:S445–54. 10.2105/AJPH.2013.301704r PMC3935673 24328646

[46] Breslow N. A generalized Kruskal-Wallis test for comparing K samples subject to unequal patterns of censorship. Biometrika. (1970) 57:579–94. 10.1093/biomet/57.3.579

[47] Retkute R Jewell CP Van Boeckel TP Zhang G Xiao X Thanapongtharm W. Dynamics of the 2004 avian influenza H5N1 outbreak in Thailand: the role of duck farming, sequential model fitting and control. Prev Vet Med. (2018) 159:171–81. 10.1016/j.prevetmed.2018.09.014 30314780 PMC6193140

[48] Bank W. World Bank Open Data Online. (2020). Available online at: https://data.worldbank.org/indicator/AG.LND.ARBL.HA.PC (accessed 12 May, 2020).

[49] Statistics Korea. Livestock Statistics Survey Korea. (2015). Available online at: http://kosis.kr/statisticsList/statisticsListIndex.do?menuId=M_01_01&vwcd=MT_ZTITLE&parmTabId=M_01_01?menuId=M_01_01&vwcd=MT_ZTITLE&parmTabId=M_01_01&parentId=F#SubCont (accessed 12 May, 2020).

[50] Kim WH An JU Kim J Moon OK Bae SH Bender JB. Risk factors associated with highly pathogenic avian influenza subtype H5N8 outbreaks on broiler duck farms in South Korea. Transbound Emerg Dis. (2018) 65:1329–38. 10.1111/tbed.12882 29673109

[51] OECD. Producer Incentives in Livestock Disease Management. Paris: OECD (2017).

[52] Gilbert M Slingenbergh J Xiao X. Climate change and avian influenza. Rev Sci Tech. (2008) 27:459. 10.20506/rst.27.2.1821 18819672 PMC2709837

[53] Tian H Zhou S Dong L Van Boeckel TP Pei Y Wu Q. Climate change suggests a shift of H5N1 risk in migratory birds. Ecol Model. (2015) 306:6–15. 10.1016/j.ecolmodel.2014.08.005

[54] Lowen AC Mubareka S Steel J Palese P. Influenza virus transmission is dependent on relative humidity and temperature. PLoS Pathog. (2007) 3:e151. 10.1371/journal.ppat.0030151 17953482 PMC2034399

[55] Liu C-M Lin S-H Chen Y-C Lin KC-M Wu T-SJ King C-C. Temperature drops and the onset of severe avian influenza A H5N1 virus outbreaks. PLoS ONE. (2007) 2:e191. 10.1371/journal.pone.0000191 17297505 PMC1794318

[56] Brown JD Goekjian G Poulson R Valeika S Stallknecht DE. Avian influenza virus in water: infectivity is dependent on pH, salinity and temperature. Vet Microbiol. (2009) 136:20–6. 10.1016/j.vetmic.2008.10.027 19081209

[57] Son K Kim YK Oem JK Jheong WH Sleeman J Jeong J. Experimental infection of highly pathogenic avian influenza viruses, clade 2.3. 4.4 H5N6 and H5N8, in mandarin ducks from South Korea. Transbound Emerg Dis. (2018) 65:899–903. 10.1111/tbed.12790 29266850

[58] Delamater PL Street EJ Leslie TF Yang YT Jacobsen KH. Complexity of the basic reproduction number (R0). Emerg Infect Dis. (2019) 25:1. 10.3201/eid2501.171901 30560777 PMC6302597

[59] USDA Foreign agricultural service. Status of Highly Pathogenic Avian Influenza in South Korea Seoul. (2016). Available online at: https://kr.usembassy.gov/wp-content/uploads/sites/75/2017/01/KS-1648-Status-of-Highly-Pathogenic-Avian-Influenza-in-South-Korea_12-19-2016.pdf (accessed 6 May, 2020).

[60] Kim E-T Pak S-I. The contribution of farm vehicle movements for a highly pathogenic avian influenza epidemic in 2014 in the Republic of Korea. J Prev Vet Med. (2019) 43:182–8. 10.13041/jpvm.2019.43.4.182

[61] Kwon H-i Kim E-H Kim Y-i Park S-J Si Y-J Lee I-W. Comparison of the pathogenic potential of highly pathogenic avian influenza (HPAI) H5N6, and H5N8 viruses isolated in South Korea during the 2016–2017 winter season. Emerg Microbes Infect. (2018) 7:–29. 10.1038/s41426-018-0029-x 29535296 PMC5849756

